# When the cure becomes the curse: Radiation-induced glioma of the pons in children surviving craniopharyngioma

**DOI:** 10.1093/nop/npag018

**Published:** 2026-02-27

**Authors:** Rahat Ul Ain, Hamza S Gorsi, Celine Habre, Andrea Bartoli, Sebastien Tran, Rabia A Qaiser, Laeeq Ur Rehman, Steven R Miller, Eric Bouffet, André O von Bueren

**Affiliations:** Department of Pediatric Hematology/Oncology, University of Child Health Sciences, The Children’s Hospital Lahore, Lahore, Punjab, Pakistan; Central Michigan University, Mount Pleasant, MI (HSG); Division of Hematology/Oncology, Children's Hospital of Michigan, Detroit, MI (HSG); Pediatric Radiology Unit, Radiology Division, Diagnostic Department, University Hospital of Geneva, Geneva, Switzerland; Department of Neurosurgery, University Hospital of Geneva, Geneva, Switzerland; Department of Radiation Oncology, University Hospital of Geneva, Geneva, Switzerland; Department of Pediatric Radiology, University of Child Health Sciences, The Children’s Hospital Lahore, Lahore, Punjab, Pakistan; Department of Pediatric Neurosurgery, University of Child Health Sciences, The Children’s Hospital Lahore, Lahore, Punjab, Pakistan; Department of Oncology, Wayne State University School of Medicine, Detroit; Department of Paediatrics, The Hospital for Sick Children, University of Toronto, Toronto (E.B.); Department of Pediatrics, Gynecology and Obstetrics, Division of Pediatric Hematology and Oncology, University Hospital of Geneva, Geneva, Switzerland; Cansearch Research platform for pediatric oncology and hematology, Faculty of Medicine, Department of Pediatrics, Gynecology and Obstetrics, University of Geneva, Geneva, Switzerland

**Keywords:** brain stem neoplasms, central nervous system neoplasms, child, craniopharyngioma, glioma, neoplasms, radiation-induced

## Abstract

**Background:**

The standard of care for pediatric craniopharyngioma (CP) is either complete surgical resection or limited debulking surgery followed by adjuvant radiotherapy (RT). Recent data favor the latter approach.

**Methods:**

We present a multi-center case series of three children with CP treated with subtotal surgery and adjuvant RT for CP who eventually developed radiation-induced glioma (RIG) of the pons.

**Results:**

The median age at diagnosis of CP was 6 years. Photon RT was administered in two patients and proton RT in one, at a median dose of 54 Gy delivered in 30 fractions. Pontine glioma was first identified on imaging after a median interval of 8 years from initial RT (range 4.5 - 9.75 years), incidentally in two asymptomatic patients during routine surveillance, and associated with symptoms of brainstem involvement in one. A biopsy was performed in two patients. One showed diffuse astrocytoma with *MYCN*, *PDGFRA*, and *MDM2* amplification and a novel fusion (RBD7-FLI1), while the other case had an inconclusive histopathology. Re-irradiation (re-RT) (54 Gy/30 fractions and 30 Gy/10 fractions) was applied in two patients, and one patient received bevacizumab treatment. All three patients succumbed with a median survival of 7.5 months (range 4.0–58 months).

**Conclusions:**

Radiation-induced glioma of the pons is a rare and serious complication occurring in patients previously treated by RT for childhood CP. This secondary tumor is uniformly associated with a dramatic course regardless of the treatment modalities and resources. This highlights the need for clinical vigilance and further research to prevent it.

Key Points

- Rare radiation-induced pontine glioma can occur after craniopharyngioma treatment
- Radiotherapy needs to be continually optimized for safety and efficacy
- Risks must be discussed with patients and families.

Importance of the Study

Maximum safe resection followed by radiation therapy is the preferred approach for the treatment of craniopharyngioma (CP). Our series shows that there is a potential risk of RI-pontine glioma in patients with CP. Therefore, radiation techniques with very high conformity should be preferentially used and it is important to identify patients who may be spared radiation treatment. Moreover, the risk of RI-pontine glioma should be discussed with the families/patients before deciding to use radiation therapy for the treatment of CP. The limitations of this study can be overcome by studying larger prospective datasets and advocating post-mortem biopsies for molecular classification and germline mutations. Although rare, our findings underscore the importance of heightened clinical awareness of this severe complication of the treatment. The outcomes are uniformly poor, reflecting the grave prognosis of this condition, seemingly independent of treatment modalities, resources, and surveillance.

Craniopharyngioma (CP) in children is a rare central nervous system (CNS) tumor that originates from the epithelial remnants of Rathke’s pouch, commonly at the sellar/parasellar region.^1^ It is a benign low-grade tumor (WHO Grade 1) classified into two phenotypes: adamantinomatous CP (adaCP) and papillary CP (papCP)^2^ with different genetic and epigenetic backgrounds.^3^ BRAFV600E mutations are found in papCP subgroup. In contrast, CTNNB1 mutations are exclusively detected in adaCP.^3^ The adaCP type is the predominant histopathology in the pediatric population.^4^ Currently, the favored approach combines limited surgery and adjuvant proton radiotherapy (RT). This sequential approach is associated with the best outcomes in terms of local tumor control, survival, and complication rates.^5^^,^^6^

RT plays a central role in the management of various ­pediatric CNS tumors, but also carries the risk of radiation-induced gliomas (RIGs) having a poor prognosis.^7–9^ Methylation profiling shows that RIGs primarily cluster within the GBM pedRTK1 methylation group with commonly reported molecular alterations of *TP53* mutations, *PDGFRA* amplification, and *CDKN2A/B* deletion.^10–12^ The management of RIGs is not well-defined and offers no curative potential for most patients.^13^ Diffuse intrinsic pontine glioma (DIPG) is a clinical-radiologic diagnosis carrying out a very poor outcome.^12^ The diagnosis of DIPG relies on the presence of a midline mass affecting at least 2/3 of the cross-sectional area of the basis pons at magnetic resonance imaging (MRI), in combination with symptoms and signs of cranial nerve root and brainstem involvement.^14–18^ DIPGs commonly harbor H3K27 alterations, H3 alterations being acknowledged to define diffuse midline gliomas (DMGs) according to the World Health Organization (WHO) CNS tumor classification 2021.^19^ DIPGs that do not have H3 alterations are called DMG wild-type (WT) or, more precisely, diffuse pediatric-type high-grade gliomas H3-WT and IDH-WT.^19^ Most pontine gliomas occur de novo, and RIGs of the pons are relatively rare.^7–12^

The objective of this multi-center case series is to describe the clinical course of three adolescents who had been previously treated by surgery and adjuvant RT for a childhood CP in three different institutions, and who subsequently developed RIG of the pons during the follow-up while in remission of the primary tumor.

## Methods

This study was conducted as part of a multi-institutional collaboration involving the Children’s Hospital Lahore, Pakistan, the Children’s Hospital of Michigan, USA, and the University Hospital of Geneva, Switzerland. Three patients who developed pontine glioma after treatment of CP with RT were retrospectively identified by the investigators, and data were obtained through chart review (Table 1). Written informed consent for submission and publication of these three cases, including accompanying images and clinical details, was obtained from the patients or their legal representatives, in accordance with COPE guidelines.

**Table 1.** Clinical details of all three reported cases of radiation-induced DIPG/DMG in patients with pediatric Craniopharyngioma

Case #	Age at diagnosis of CP^a^	Gender	Extent of surgery	Radiotherapy details	Age at diagnosis of RI-DIPG	Management of DIPG/DMG	Biopsy findings of DIPG	Latency period (months)	Survival since diagnosis of DIPG (months)
1.	6 year	Male	NTR^b^	3D-conformal: 54 Gy^d^/30 fr^e^	14 y	RT: 30Gy/10 fr Steroids	Not done	96 months	7.5 months
2.	6 year	Female	STR^c^	Photon, 30 fr, 50.4 Gy/28 fr EBRT^f^	15.8 y	RT: 54Gy/30 fr Avapritinib on progression	Diffuse astrocytoma	117 months	58 months
3.	9.25 year	Female	STR	Proton IMPT^d^ 54 Gy/30 fr of 1.8 Gy	17.5 y	Bevacizumab (No RT)	Inconclusive	54 months	22 months (4 months) after fulfilling the radiologic criteria for DIPG

a Craniopharyngioma.

b Near-total resection.

c Subtotal resection.

d Grays.

e Fractions.

f External beam radiotherapy.

d Intensity modulated proton therapy.

## Results

### Case 1

**Figure 1.**
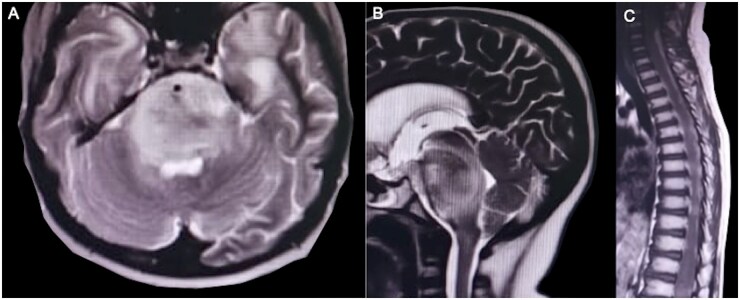
(A-C) Magnetic Resonance Imaging of Case 1 (14-year-old patient, 8 years after adjuvant RT for CP). The quality of the selected images is related to the copy of the images from film prints. Axial T2-weighted image shows an expansive mass infiltrating the entire cross-section of the basis pontis, engulfing the basilary artery in the prepontine cisterna, and extending to the middle cerebellar peduncles, fulfilling the imaging criteria of DIPG (A). Sagittal T2-weighted shows the cranial extension of the mass to the midbrain with mass effect of the cerebral aqueduct and secondary obstructive hydrocephalus with visible dilatation of the third ventricle (B). Follow-up MRI performed in the setting of acute paraplegia, i.e., 5 months after completion of re-irradiation, demonstrating leptomeningeal dissemination on sagittal contrast-enhanced T1-weighted images of the spine (C).

A 14-year-old boy presented with complaints of headache and iterative vomiting over one month, gait instability, and slurred speech worsening over 1 week. Eight years before, he had undergone near-total surgical excision of an adaCP followed by 3D-conformal radiation therapy (54 Gy/30 fractions). On admission, his Glasgow Coma Scale (GCS) was 15/15 with a Lansky Performance Score (LPS) of 20/100. His height was below the 3^rd^ percentile for his age. The patient had ataxia and dysmetria on examination. A brain MRI with contrast-media injection showed an expansive mass occupying the entire cross-sectional area of the basis pontis, and extending to the midbrain, as demonstrated by the hyperintense signal extension on T2-weighted images with moderate obstructive hydrocephalus (Figure 1A and B). The radiological features, along with the typical clinical manifestations, were consistent with the diagnosis of DIPG/pontine glioma (Figure 1). Tissue biopsy was not deemed necessary to confirm the diagnosis. Considering the previous RT, palliative photon RT was administered with a delivery dose of 30 Gy in 10 fractions. Under therapy, his symptoms resolved gradually, and his LPS improved from 20 to 80/100 within four months after completion of RT. However, after eight months of RT, the patient developed severe back pain refractory to multiple analgesic medications. A month later, he developed sudden paraplegia, thought to be related to diffuse spinal leptomeningeal dissemination as demonstrated by contrast-enhanced MRI of the brain and spine, despite the favorable response of the secondary tumor of the pons (Figure 1C). Comfort and supportive care for the patient and psychosocial support for the family were offered. He succumbed 7.5 months after his initial presentation.

### Case 2

A 6-year-old female presented with an evolving headache over six weeks and left eye medial deviation for two days. MRI brain revealed a solid/cystic mass in the sellar/suprasellar region with heterogeneous enhancement and obstructive hydrocephalus. She underwent subtotal resection, and the pathology was consistent with adaCP. She received adjuvant RT to a total dose of 50.4 Gy, delivered in 28 fractions of 1.8 Gy using Intensity Modulated Radiation Therapy (IMRT). Post-operatively, she developed pan-hypopituitarism, requiring desmopressin, hydrocortisone, levothyroxine, and growth hormone substitution. Recurrence of CP was identified during routine MRI surveillance 22 months after the initial diagnosis. She underwent a second surgery where a gross total resection was achieved, and the pathology confirmed a recurrent CP.

Almost 10 years after the initial presentation, while the patient was asymptomatic and continuously monitored by MRI, an initially ill-defined T2-hyperintense lesion appeared within the basis pontis and progressively increased in size over almost 9 months. A biopsy of this expanding lesion was performed, and the histology of the lesion was compatible with a diffuse astrocytoma. On molecular sequencing, the tumor was negative for *H3K27M* or *IDH* mutation but showed *MYCN*, *PDGFRA,* and *MDM2* amplification, and a novel RBD7-FLI1 fusion, previously so-called DMG-WT. According to the most recent 2021 WHO classification of CNS tumors, this tumor is recognized as a diffuse pediatric-type high-grade glioma H3 wild type, IDH wild type. She received photon beam radiation (54 Gy in 30 fractions) and tolerated it well without any complications. The family refused the option of temozolomide treatment or enrollment in any clinical trial at that time.

**Figure 2.**
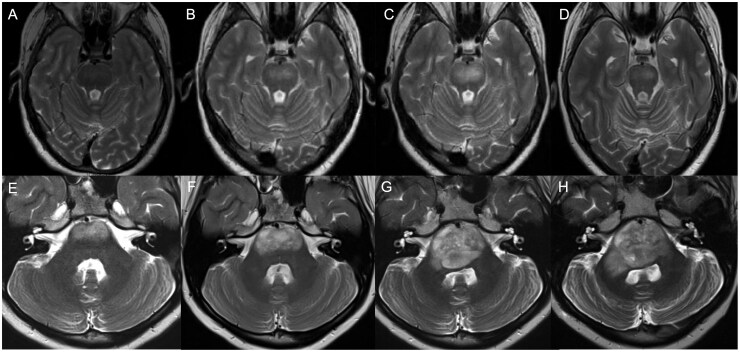
(A-D) Magnetic Resonance Imaging of Case 2 (15.8-year-old patient, 10 years after adjuvant RT for CP). Serial axial T2-weighted images one year earlier (A), and at the first time a hyperintense ill-defined lesion centered in the basis pontis was identified on follow-up imaging (B). Eight months later, the pontine lesion had increased in size, reaching 2/3 of the cross-sectional area of the basis pontis, although remaining relatively homogenous (C), therefore leading to a biopsy that enabled to reach the diagnosis of RIG. At that time the patient was asymptomatic. MRI performed after re-irradiation shows a decrease in size and intensity of the basis pontine lesion due to favorable response to RT (D). (E-H) Magnetic Resonance Imaging of Case 3 (16-year-old patient, 3 years after adjuvant RT for locally recurrent CP). Serial axial T2-weighted images during surveillance of the asymptomatic patient. Initial faint hyperintense lesion within the basis pontis (E). One year later, the ill-defined T2-hyperintense lesion of the basis pontis had increased in size with subtle ventral growth within the prepontine cisterna (F). One year and a six months later, the lesion became increasingly heterogeneous, occupying the entire surface of the basis pontis with ventral exophytic growth engulfing the basilary artery, these findings highly suggestive of DIPG despite the absence of symptoms (G). Two months later after the radiologic diagnosis, 1 year and 8 months after the pontine lesion had appeared, the mass showed further extension into the right middle cerebellar peduncle, coinciding with the onset of symptoms and prompting biopsy (H).

She started developing headaches, imbalance, and difficulty with swallowing and nasal speech 4.5 years after the diagnosis of RI pontine glioma. At the time the patient became symptomatic, MRI showed the progression of the abnormal T2-hyperintense ill-defined lesion from now on occupying 2/3 of the cross-sectional area of the basis pontis (Figure 2A-D). The patient was thus started on a PDGFRA inhibitor (avapritinib) and concurrent best supportive/palliative care. Progressive worsening of symptoms and disability led the patient to pass away 58 months from the initial diagnosis of RIG of the pons and 43 months after completion of re-irradiation RT.

### Case 3

A 9-year-old female patient presented with fatigue, obesity, and visual disturbances, prompting further diagnostic evaluation. A cranial MRI revealed a cystic suprasellar mass, raising the concern for a CP that was confirmed by a biopsy as an adaCP. From an endocrinologic perspective, the tumor was associated with panhypopituitarism, requiring complete hormonal replacement therapy. She further underwent partial surgical resection of the suprasellar tumor, followed by a second surgery three years later due to local recurrence and mass effect. This second intervention at the age of 12 years was combined with adjuvant IMRT (total dose: 54 Gy in 30 fractions of 1.8 Gy, clinical target volume (CTV) = gross tumor volume (GTV) + 5mm, planning target volume (PTV) = CTV + 4mm). The patient underwent regular follow-up with serial MRI imaging and multidisciplinary clinical monitoring, including assessments by neurology, ophthalmology, and endocrinology.

Three years after completion of RT, when the patient was 15 years old and was otherwise asymptomatic, routine surveillance MRI revealed the new occurrence of a faint and ill-defined T2-hyperintense lesion in the basis pons, within an area that had received between 30 and 50 Gy (Figure 2E-H; Supplementary Material, Figure S2). Intensive imaging follow-up was initiated to monitor the lesion, for which the differential diagnosis included delayed radiation-induced injury versus RIG. The basis pontis lesion steadily increased in size over 1.5 years, reaching the entire cross-sectional area of the basis pontis, expanding to the middle cerebellar peduncles, and growing ventrally with partial engulfment of the basilar artery, thus fulfilling all the imaging criteria for DIPG (Figure 2E-H). At the time the radiologic diagnosis RIG became unequivocal, the patient remained asymptomatic. However, 2 months later, that is, 20 months after the pontine lesion had appeared for the first time (Figure 2E-H), the 17.5-year-old adolescent developed ataxia and cranial nerve palsies. Follow-up was intensified, and a biopsy was offered to confirm the diagnosis, but it failed to yield conclusive results. The family declined re-irradiation; therefore, bevacizumab therapy was started to reduce the need for steroid treatment, and best supportive care was offered to provide palliation for the patient. Once the clinical picture of DIPG had fully developed, the patient’s condition deteriorated rapidly, and she passed away within 4 months.

## Discussion

This is the first report specifically addressing RIG of the pons arising after CNS radiation for craniopharyngioma (CP). The past radiation therapy details (Supplementary Material) confirmed that in cases 2 and 3 the brainstem was exposed to RT doses approximatively between 30 and 50 Gy (Supplementary Material, Figures S1 and S2). Among the three cases of post-radiation pontine glioma in our case series, two were asymptomatic, diagnosed on routine surveillance MRI, and the third patient presented with new onset of symptoms, and a diagnosis of pontine glioma was confirmed with MRI. Interestingly, asymptomatic patients who were diagnosed on routine surveillance had longer survival (58 months and 22 months) as opposed to the case-identified based on symptoms (7.5 months), showing the benefit of routine surveillance post-radiation. The sustained tumor control observed with patient 2, who was treated with RT early after the diagnosis of RIG of the pons, is interesting and suggests that early intervention can be beneficial in this context. It is, however, not clear if this difference in outcome was due to the molecular features of this secondary tumor or early treatment. However, patient 2 had classic molecular features of RIG, such as PDGFRA amplification, which harbors poor outcomes and aggressive disease, and an unfavorable prognosis.^19^ The pontine location of the secondary tumors in our case series made them very challenging to treat, as the tumors in this location are not amenable to surgical resection.

Over the last two decades, the management of CP has moved to a limited resection followed by radiation therapy, with no evidence of a detrimental effect on survival and a clear benefit in terms of cognitive and behavioral outcome.^20^ However, very rarely, long-term follow-up papers have suggested that RIG of the pons may occur after RT.^21^ Our series shows that there is a potential risk of RIG of the pons in patients with CP. Therefore, radiation techniques with very high conformity should be preferentially used to protect vulnerable organs like the brainstem, and it is important to identify patients who may be spared radiation treatment. Moreover, the risk of RIG of the pons should be discussed with the families/patients before deciding to use radiation therapy for the treatment of CP. The emerging targeted therapies like Tovorafenib (pan-RAF-kinase inhibitor), Binimetinib (MEK Inhibitor), and Tocilizumab (Systemic IL-6 Receptor Antagonist) hold promises in treating pediatric craniopharyngioma without RT and are currently under phase 2 clinical trials.^22–24^

Our study has several limitations. It is a retrospective study with a small number of patients. Longer survival in the 2 asymptomatic patients identified on routine surveillance may reflect lead-time bias rather than the effect of early intervention. Larger cohorts need to be studied to better understand the clinical pattern of this rare presentation and to investigate the dose–volume correlations and their relationship to time in order to optimize future radiotherapy treatment for CP. We advocate considering post-mortem biopsy and germline analyses in all cases of RIG of the pons to explore molecular features and potential cancer predisposition syndromes.

## Conclusion

Although RIG of the pons is rare in patients treated with cranial irradiation for CP, our findings underscore the importance of heightened clinical awareness of this severe complication of the treatment. The outcomes are uniformly poor, reflecting the grave prognosis of this condition, seemingly independent of treatment modalities, resources, and surveillance.

## Supplementary Material

npag018_Supplementary_Data

## Data Availability

The data underlying this article are available in the article and in its online supplementary material.
