# Supplementary material for: When the cure becomes the curse: Radiation-induced glioma of the pons in children surviving craniopharyngioma
Source: Neurooncol Pract. 2026 Feb 27;13(4):816–22. doi: 10.1093/nop/npag018 (PMC13365136; doi:10.1093/nop/npag018)
Supplement: npag018_Supplementary_Data [file npag018_supplementary_data.zip › Supplementary Material AvB_hg 19 Feb 2026 AvB.docx]

**Supplementary Material: NOP-D-25-00258R1**

**Radiation Therapy (RT) Details**

**Case 1:** The RT details for Case 1 were not available.

**Case 2:** The gross tumor volume (GTV) included the postoperative tumor bed and residual disease. The planning target volume (PTV) was generated by adding a 1-cm margin to the GTV. A total dose of 50.4 Gy was delivered in 28 fractions of 1.8 Gy using Intensity Modulated Radiation Therapy (IMRT) with 6 Megavoltage (MV) photons (Supplementary Figure S1)


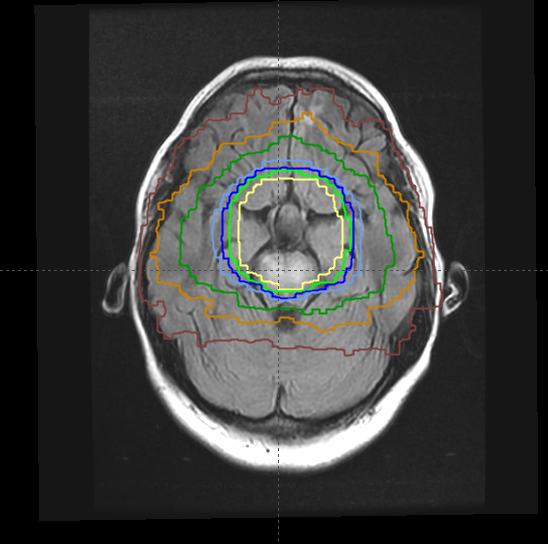

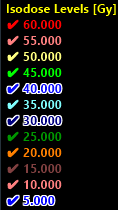


Supplementary Figure S1: Magnetic resonance imaging of Case 2 (15.8-year-old patient, 10 years after adjuvant radiotherapy for craniopharyngioma). An axial T1-weighted image shows a pontine lesion, leading to biopsy and diagnosis of radiation-induced glioma. Photon isodose distributions from the initial craniopharyngioma treatment are shown. The isodose color legend is displayed in the right panel.

**Case 3:** The proton therapy treatment of case 3 was comparable to the series reported before by Merchant et al. ^6^. In brief, the gross tumor volume was defined as the postoperative tumor bed and residual tumor, and the clinical target volume included an anatomically defined margin of 0.5 cm surrounding the gross tumor volume. The planning target volume was a geometric margin of 0.4 cm surrounding the clinical target volume.

The clinical target volume was intended to include subclinical microscopic disease. The planning target volume was meant to account for variation in daily treatment, beam uncertainties, and aperture design. Proton-specific uncertainties were accounted for in the design of each proton beam. The prescribed total dose was 54 Gy (relative biological effect [RBE]) using conventional fractionation of 1·8 Gy (RBE) per day. In Case 3, the RIG developed in an area that had received a radiation dose of 30–50 Gy (Supplementary Figure S2).

1. Merchant TE, Hoehn ME, Khan RB, et al. Proton therapy and limited surgery for paediatric and adolescent patients with craniopharyngioma (RT2CR): a single-arm, phase 2 study. *Lancet Oncol.* 2023;24(5):523-534.


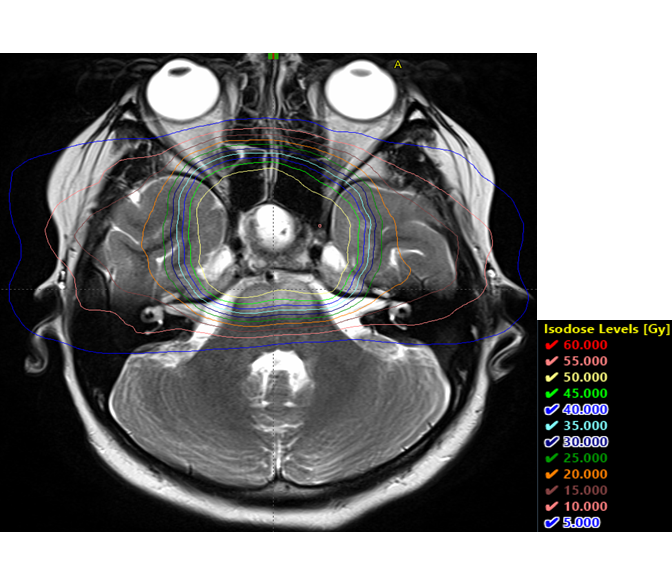
 Supplementary Figure S2: Magnetic Resonance Imaging of Case 3 (16-year-old patient, 3 years after adjuvant proton therapy for locally recurrent CP). Serial axial T2-weighted images during surveillance of the asymptomatic patient. Initial faint hyperintense lesion within the basis pontis, with the Proton isodoses superimposed. The isodose line color legend is shown in the top right panel.

**Summary of RT findings of all cases**

The median age at diagnosis of CP was 6 years, with two females and one male. All three CP patients underwent subtotal resection followed by RT with photons (n=2) and protons (n=1) at a median dose of 54 Gy/30 fractions. Radiation-induced (RI) pontine gliomas were diagnosed after a median duration of 8 years after initial radiation therapy (range 4.5- 9.75 years). Two cases were diagnosed during surveillance imaging, and a third case was diagnosed after the development of new symptoms of a brainstem lesion. A biopsy was performed in two patients. One showed diffuse astrocytoma with MYCN, PDGFRA, and MDM2 amplification and a novel fusion (RBD7-FLI1), while the other case had an inconclusive histopathology. Re-RT was given in two cases (54 Gy/ 30 fractions and 30 Gy/10 fractions), while the third case refused re-RT and received bevacizumab treatment. All three cases succumbed to RI- pontine glioma with a median survival of 7.5 months (Range 4.0 – 58 months).
